# Tough decisions on essential medicines in 2015

**DOI:** 10.2471/BLT.15.154385

**Published:** 2015-03-12

**Authors:** Nicola Magrini, Jane Robertson, Gilles Forte, Bernadette Cappello, Lorenzo P Moja, Kees de Joncheere, Marie-Paule Kieny

**Affiliations:** a World Health Organization, avenue Appia 20, 1211 Geneva 27, Switzerland.

In 1977, the World Health Organization (WHO) published its first Model List of Essential Medicines.^1^ This year, the Expert Committee for the Selection and Use of Medicines will consider requests to include high-cost medicines for cancer, hepatitis C, multidrug-resistant tuberculosis and new oral anticoagulants on the model list. These applications challenge perceptions of essential medicines and raise questions about how to address issues of cost and affordability for countries when making decisions at the global level.

Essential medicines are those that satisfy the priority health-care needs of the population.^2^^,^^3^ In addition to public health relevance, essential medicines are selected with due regard to evidence on efficacy and safety, and comparative cost–effectiveness. Methods for the selection of essential medicines were reviewed by WHO’s Executive Board in 2001.^3^ The Executive Board specifically noted that absolute treatment cost should not be a reason to reject a proposed addition to the model list if criteria for benefit and public health relevance are met. In practice, affordability has been changed from a precondition for listing an essential medicine to a consequence that must be managed after the decision to list.^2^

Yet considerations of costs and budget impact – along with registration status of the medicine, the feasibility of its use in various clinical settings and the need for monitoring – must also influence global decision-making. A single approach to determining the affordability of new medicines is unlikely to succeed. Enabling access to cost-effective, yet potentially unaffordable, therapies will require particular consideration by the expert committee and new and better-coordinated actions at a global level. Tools such as incremental cost-effectiveness analysis can inform country-level decisions on adding a new medicine to a formulary or a reimbursement list. However, these methods do not address the issues of budget impact or affordability of a medicine. Experience suggests that in the absence of competition, options may be limited. Other tools such as those of WHO-CHOICE (CHOosing Interventions that are Cost–Effective) may help national policy-makers decide what is a reasonable price to pay for a medicine.^4^ The challenge is to provide access to effective medicines without creating ad hoc vertical programmes and, at the same time, to avoid diverting funds from other important health-care services. Regional pooled procurement mechanisms, price controls, dedicated funding for specific needs, differential pricing and licence agreements can be effective ways of managing costs. 

Previous expert committees have recognized the message that comes with identifying a medicine as essential. In some cases, medicines have been included in the core list to underscore their importance, for example, antiretrovirals in 2002.^5^ In other cases, the model list has been used to stimulate the entry of new manufacturers for products that are not widely available, such as with zinc sulfate in 2005 and rectal artesunate in 2009. Inclusion of effective but expensive medicines in the model list may also focus the attention of all stakeholders on the need to increase affordability and access to essential medicines.

In 2013, the expert committee defined public health relevance to encompass overall incidence and prevalence of diseases as well as diseases that are specific to certain regions and diseases that are uncommon but for which there are effective medicines.^6^ This broader framework allows the committee to include medicines for comparatively rare conditions such as leukaemia. The committee’s main criteria for inclusion in the list are the magnitude of clinical benefit and a favourable risk–benefit profile determined through a systematic method of evidence synthesis and appraisal.^7^

Estimates of the magnitude of the benefit are particularly pertinent for medicines for cancer, given the small gains in life expectancy offered by some new and expensive treatments. Previous expert committee decisions confirm the preference for listing treatments that offer cure or effective disease management over those that offer only marginal benefit. There have been calls for changes to regulatory assessments to ensure that only medicines offering clinically relevant improvements in cancer survival, or large clinical benefit, receive marketing approval.^8^^,^^9^ The American Society of Clinical Oncology proposes minimum benefit thresholds for the design of clinical trials,^10^ while the European Society for Medical Oncology is working to develop tools to assess the clinical benefits of cancer treatments. It is yet to be determined whether the expert committee will suggest a minimum threshold of benefit for cancer medicines, but prioritization according to the magnitude of benefit is a guiding principle that can assist countries in developing their national essential medicines lists.

Two novel agents against tuberculosis, bedaquiline and delamanid, achieved regulatory approval based on promising, though limited, data from clinical trials. The trials used sputum culture conversion after the first few months of treatment as a surrogate marker of outcome. WHO issued interim guidance on the use of bedaquiline and delamanid for multidrug-resistant tuberculosis,^11^^,^^12^ due to the public health relevance and severity of the condition and the lack of alternative treatment options. Mechanisms will be needed to ensure access to – and safe use of – these medicines while further evidence on efficacy and safety from phase III clinical trials is generated. 

The model list uses a classification of core and complementary medicines. This does not imply that only core medicines should be procured by the public system, while complementary medicines are optional. The core list includes the minimum medicines needed for a basic health-care system, while the complementary medicines list includes medicines for diseases that require more specialized diagnostic or monitoring facilities, medical care, and training.^6^ The model list can be adapted to meet national needs and health priorities. Its principles and approaches are equally relevant to high-, middle-, and low-income countries and have increasing relevance as countries implement medicines benefits packages as part of universal health coverage. The next expert committee (in its April 2015 meeting) will need to consider how to realize the global health benefits of new medicines for which affordability is a major issue.
